# Aerobic, Strengthening, and Balance Activities Performed by Community-Dwelling Older Adults

**DOI:** 10.1093/geroni/igab046.1310

**Published:** 2021-12-17

**Authors:** Mariana Wingood, Bonnell Levi, Nancy Gell

**Affiliations:** 1 University of Vermont, University of Vermont, Vermont, United States; 2 University of Vermont, Burlington, Vermont, United States

## Abstract

Little is known about whether older adults meet the recommended physical activity (PA) guidelines, including aerobic, strength, and balance components. Given this gap, we examined self-report PA data from 1,352 older adult participants of the Adult Changes in Thought (ACT) study. We classified participants as meeting some components, meeting the full guidelines, or being insufficiently active. Multinomial regression was used to identify factors associated with meeting PA guidelines. Despite performing 9.5 hours of weekly PA, only 11% met the full guidelines, 13% met the aerobic, and 26% met the balance or strength recommendations. Increasing age and body mass index, needing assistance with instrumental daily activities, heart disease, and low income were associated with decreased odds of meeting PA guidelines. Older adults primarily perform aerobic PA and lower intensity PA with fewer participating in strength and balance activities. Interventions targeting strength, balance, and higher intensity PA should be developed.

